# Outcomes of Percutaneous Portal Vein Intervention in a Single UK Paediatric Liver Transplantation Programme

**DOI:** 10.1007/s00270-017-1792-0

**Published:** 2017-09-14

**Authors:** Ravi Patel, Jeevan Mahaveer, Nasim Tahir, Sanjay Rajwal, Patricia McClean, Jai V. Patel

**Affiliations:** 1 School of Clinical Medicine, University of Cambridge, Cambridge, UK; 2 Departmentt of Radiology, Leeds Teaching Hospitals NHS Trust, Leeds, UK; 3 Department of Paediatric Hepatology, Leeds Teaching Hospitals NHS Trust, Leeds, UK

**Keywords:** Portal vein, Liver transplantation, Paediatric, Angioplasty, Stent

## Abstract

**Introduction:**

Percutaneous transluminal angioplasty (PTA), with or without stent placement, has become the treatment of choice for portal vein complications (PVC) following liver transplantation. We aimed to assess long-term outcomes of intervention in paediatric transplant recipients, in a single institution.

**Materials and Methods:**

227 children received 255 transplants between November 2000 and September 2016. 30 patients developed PVC of whom 21 had percutaneous intervention. Retrospective clinical and procedural outcome data on these 21 patients were collected.

**Results:**

21 patients, with median age 1.7 years (range 0.4–16.2), underwent 42 procedures with PTA with or without stenting. 36 procedures were for PV stenosis and 6 for PV thrombosis. Treatment was with primary PTA, with stenting reserved for suboptimal PTA result or restenosis within 3 months. 28 procedures were performed with PTA and 13 with stenting. Technical success (>50% reduction in mean pressure gradient, absolute pressure gradient ≤4 mmHg or venographic stenosis <30%) was achieved in 41 procedures. Failure to recanalise a thrombosed PV occurred in 1 procedure. There were no major procedural complications. Patients were followed-up with serial Doppler ultrasound surveillance. Kaplan–Meier estimated median primary patency was 9.9 months, with primary-assisted patency of 95% after median follow-up of 45.5 months (range 11.1–171.6).

**Conclusion:**

With regular surveillance, excellent patency rates can be achieved following percutaneous intervention for PVC post-paediatric liver transplantation.

## Introduction

Portal vein complications (PVC) following paediatric liver transplantation have an incidence of <3–14% [1] and can lead to graft failure resulting in significant morbidity and mortality [2–4]. Risk factors for PV thrombosis (PVT) or PV stenosis (PVS) include age under 1 year old at first transplant, lower body weight, preexisting portosystemic shunts with decreased PV flow [5–7] and hypoplasia of the PV, commonly seen in patients with biliary atresia [8–10]. Clinical manifestations of PVC are those of portal hypertension, including new onset ascites, splenomegaly and variceal bleeding. Abnormal liver function tests and even liver failure may occur with early PVT post-liver transplant. Some patients are asymptomatic [4].

Percutaneous transluminal angioplasty (PTA) with or without stent placement, first reported by Raby in 1991 [11], has become the primary treatment option for PVC following liver transplantation. The surgical alternative would be either reconstruction of the portal vein, placement of an interposition graft (from the recipient living donor or cadaveric donor) or use of a mesenterico-left portal shunt (so-called meso-Rex shunt). In a small child with a post-operative abdomen, a surgical approach can be challenging.

In this paper, we report the midterm and long-term technical and clinical outcomes of PV intervention from a single paediatric transplant centre.

## Materials and Methods

### Patients

Patients under 18 years old, who underwent liver transplantation at our institution between November 2000 and September 2016, were identified retrospectively from a local paediatric transplant database.

Post-transplantation ultrasound Doppler (USD) was performed at days one, two, three, five and seven, months three and twelve, and thereafter annually. PVC was suspected at follow-up if there were clinical signs and symptoms of portal hypertension, or if on USD there was a velocity ratio of three or more, absolute velocity of greater than 200 cm/s, or no flow. In selected patients suspected of PVT (non-visualisation of the main PV or PV conduit, together with low-velocity intrahepatic PV flow), additional CT or MRI was performed prior to intervention to confirm the diagnosis and determine the extent of thrombosis. Patients suspected of having PVC underwent portal venography and pressure measurements, with percutaneous intervention when indicated. If PVT occurred within 2 weeks post-transplantation, surgical anastomotic revision was the preferred option.

Retrospective outcome data, on those who developed PVC and underwent percutaneous intervention, were collected from the radiology information system, and from electronic and hard copy patient records. Demographic information, technical success of the procedure, clinical success, procedural complications, primary and primary-assisted patency and current patient status were determined. Follow-up data were collected up to and including 15th July 2017.

### Procedural Technique

Informed consent was obtained from parents/guardians and where appropriate the patients, for all procedures. All procedures were performed under general anaesthesia. The exact procedural technique has been adapted over time due to changes in available consumables and devices. The following is a description of our current technique.

**Fig. 1:**
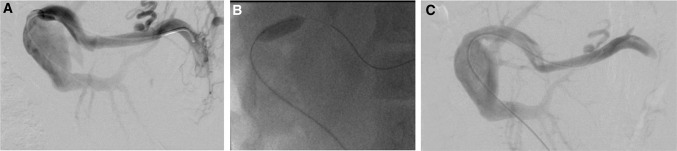
Portal vein PTA procedure from a 9-year-old male with PVS. **A** Initial venography after crossing the lesion demonstrates a focal high-grade PV stenosis at the surgical anastomosis, and pressure gradient of 3 mmHg **B** PTA balloon inflated across the stenosis. **C** Venography following PTA shows a markedly improved appearance, with no residual stenosis, and pressure gradient of 0 mmHg. *PTA* percutaneous transluminal angioplasty, *PVS* portal vein stenosis

Ultrasound guided percutaneous PV access is gained using a micropuncture access set (Cook Medical Inc, Limerick, Ireland), and a 5F arterial sheath is inserted. The stenosis is crossed with a Cobra 2 or Berenstein catheter (Cordis, Baar, Switzerland) and angled glide wire (Terumo UK Ltd, Bagshot, UK). Where difficulty in crossing the lesion is experienced either arterioportography (*n* = 1) or trans-splenic puncture (*n* = 1) are performed to provide more anatomical detail and assist in traversing the lesion. Portal venography (Fig. 1A) and pressure measurements are taken. If a significant stenosis is confirmed, PTA is performed (Fig. 1B). Heparin 75 IU/Kg is administered. The balloon diameter is based on the pre-stenotic PV diameter, inflated to between nominal and rated burst pressure, for 1 min. Repeat pressure measurements and venography are then performed to confirm success (Fig. 1C). Stenting is reserved for patients with a residual stenosis or significant pressure gradient following prolonged balloon inflation for 2–3 min, or patients who have early restenosis within 3 months. Stents are placed eccentrically across the lesion minimising stent coverage of the recipient PV. Finally, a gelatin sponge pledget is used to plug the transhepatic tract. Unless contraindicated, patients are placed on a single antiplatelet agent (Aspirin) long-term. Post-procedure anticoagulation with Heparin or Warfarin is not routine.

### Follow-Up

Patients having intervention had clinical and DUS follow-up, along with serum albumin levels and platelet count, at the following intervals: pre-discharge, at 3, 6, 12, 18, 24 months and then annually.

### Definitions

Technical success was defined as having at least one of the following apply: a pressure gradient ≤4 mmHg; ≥50% drop in pressure gradient from baseline and <30% residual venographic stenosis.

Clinical success of each procedure was assessed using four separate indicators: reduction in complications of portal hypertension, defined as ascites, variceal bleeding, splenomegaly with moderate thrombocytopenia (platelet count <100 × 10^9^/L), hepatorenal syndrome, or hepatopulmonary syndrome; reduction in spleen size measured on ultrasound; increased platelet count and increased serum albumin. For each indicator, results were compared from just prior to the procedure to the first result at least 3-month post-procedure.

Patency is reported as primary and primary-assisted patency, according to Society for Vascular Surgery (SVS) reporting standards [12]. Primary patency was defined as time from primary intervention to first imaging diagnosis of either restenosis or thrombosis. Primary-assisted patency was defined as time from primary intervention to first imaging diagnosis of thrombosis.

Complications were graded according to SVS reporting standards (mild, moderate and severe) [12] and Cardiovascular and Interventional Radiological Society of Europe (CIRSE) standards (grade 1–6) [13].

### Statistical Analysis

Two-tailed Wilcoxon signed-rank tests were used to evaluate differences between paired data. *p* < 0.05 was considered statistically significant. The Kaplan–Meier method was used to determine the cumulative primary and primary-assisted patency. Microsoft Excel 2016 and IBM SPSS Statistics 22 were used for data collection and statistical analysis, respectively.

## Results

### Demographics

Between November 2000 and September 2016, 227 paediatric patients (aged < 18 years) received 255 liver transplants at our institution. Median age at transplantation was 3.3 years (range 0.1–17.8). Post-transplantation follow-up identified 30 patients with PVC, 29 transplanted at our institution and one elsewhere: Seven had operative PV reconstruction, two had no treatment and 21 had percutaneous intervention.

Those with PVC transplanted at our institute (*n* = 29) were younger (median age 0.9 vs. 3.3 years) and of lower weight (median weight 7.1 vs. 14.0 kg) than our overall transplant population, and a greater proportion had biliary atresia as their indication for transplant (65.5 vs. 30.8%). Table 1 summarises the demographics of the 21 patients who underwent percutaneous intervention for PVC.

**Table 1:** Demographics of patients with portal vein complications undergoing percutaneous intervention

Characteristics	Values (number of patients unless stated otherwise)
Sex	
Male	10
Female	11
Age at first intervention	
Range	0.3–16.2 years
Median	1.7 years
Weight at first intervention	
Range	5.5–51.2 kg
Median	9.6 kg
Time from transplantation to first intervention	
Range	2.3–182.9 months
Median	9.0 months
Presentation prior to the 42 procedures	
Ascites	1
Variceal bleeding	4
Splenomegaly	30
Platelet count ≤150 × 10^9^/L	12 (4 patients with missing data)
Hepatopulmonary syndrome	1
Graft dysfunction	0
Type of transplant	
Cadaveric/living donor	12/9
Left lateral segment	19
Segment II only	1
Whole liver	1
Indication for transplantation	
Biliary atresia (with Kasai procedure)	14 (10)
Progressive familial intrahepatic cholestasis	1
Alagille syndrome	1
Hepatoblastoma	1
Juvenile Xanthogranulomatosis	1
Neonatal liver failure (secondary to herpes simplex virus)	1
Cholestatic liver disease (not specified)	1
Primary graft non-function (PNF) (underlying diagnosis of biliary cirrhosis with cholangitis)	1

### Technical Success

**Fig. 2:**
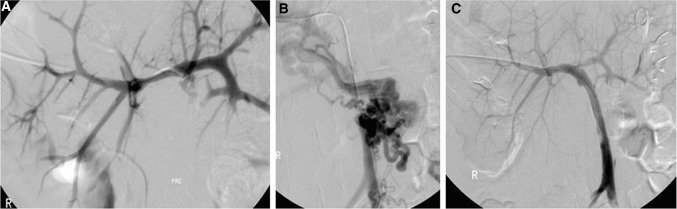
A 16-year-old female with portal vein thrombosis presented with hepatopulmonary syndrome. She had a whole graft liver transplant at the age of 1 year for biliary atresia. **A** Initial portal venography following percutaneous right PV access demonstrates patent intrahepatic PV branches with an intact PV bifurcation. **B** Following traversal of the occluded PV, venography shows occlusion extending from the main SMV trunk, with an occluded PV confluence and an associated large portal cavernoma. **C** After deployment of overlapping self-expanding stents, there is in-line PV flow from the SMV into the liver and the cavernoma is no longer seen to perfuse. Small volume non-occlusive thrombus is noted at the origin of the left portal vein, adjacent to the stent. This resolved following 48 h of therapeutic heparinisation. There was resolution of the hepatopulmonary syndrome post-procedure and the patient remains clinically well at 9 years’ follow-up. *PV* portal vein, *SMV* superior mesenteric vein

42 interventions were performed by four consultant interventional radiologists (34 by authors JP and NT), 36 for PVS and six for PVT, with a median of two per patient (range 1–5). Of the six instances of PVT, one was associated a large portal cavernoma (Fig. 2), two with smaller hilar portal collaterals and large varices, one with large oesophageal varices alone and in two cases, no cross-sectional or venographic imaging of the extrahepatic portal/splanchnic veins was available. Technical success was achieved in 41 of 42 interventions (97.6%). In one case, there was failure to recanalise a thrombosed PV. This patient underwent re-transplantation 12 months later for long-standing recurrent cholangitis, unrelated to the PVT.

28 of 41 procedures required PTA alone (with 6–10 mm balloons). 13 procedures (11/21 patients) required stent insertion (7–12 mm stents; eight Palmaz Blue, three Wallstent, one Luminexx and one Protégé) (Cordis, Baar, Switzerland; Boston Scientific, Clonmel, Ireland; BARD GmbH/Angiomed, Karlsruhe, Germany and Medtronic Ltd, Watford, UK). Four stenting procedures were for poor PTA result, three for PVT plus two recurrences in one patient and one for PVT in another patient. Nine patients had single stent procedures for early restenosis, seven at their second procedure (one subsequently developed in-stent restenosis requiring PTA) and two at their third. Where data were available (*n* = 28), there was a recorded median fluoroscopy time of 10.3 min (range 2.5–47.1), dose area product (DAP) of 258 cGy/m^2^ (range 35–3831) and overall procedure time (time from patient arrival in the department to completion of the procedure) of 135 min (range 60–280).

Pre-intervention and post-intervention pressure gradients were measured in 34 of 41 procedures, with a significant fall in the median gradient from 10.5 mmHg (range 2–22) pre-intervention to 2.0 mmHg (range 0–10) post-intervention (*p* < 0.001). The patient with a pre-procedure pressure gradient of 2 mmHg had a venographic stenosis of 63%, and therefore, fulfilled the criteria for intervention.

### Procedural Complications

There was one mild/grade 1 complication. This patient with PVT developed a small groin haematoma following an arterioportogram, which resolved without active intervention.

There were two moderate/grade 3 complications. Each patient had a focal, small, non-occlusive thrombus and brisk flow within an intrahepatic PV branch at the end of the procedure. They were commenced on therapeutic heparin post-procedure for 48 h and 14 days, respectively. Neither had residual thrombus on ultrasound pre-discharge and at 14 days.

### Clinical Success

Six procedures had no 3-month follow-up, five due to re-intervention within 3 months and one technical failure. These were, therefore, excluded from this analysis. Incomplete availability of paired pre/post-intervention data for splenomegaly with platelet count <100 × 10^9^/L excluded a further four patients for this indicator.

Seven procedures (six patients) were associated with complications of portal hypertension pre-intervention: two splenomegaly with platelet count <100 × 10^9^/L, one ascites, four variceal bleeding, one hepatorenal syndrome. All had resolved by 3 months.

There was significant reduction in spleen size (10.5 to 9.8 cm, *p* = 0.032) and significant increase in platelet count (191 to 204 × 10^9^ g/L, *p* = 0.022), but no significant change in serum albumin (41 to 42 g/L, *p* = 0.444). Although platelet count increased, thrombocytopenia (platelet count <150 × 10^9^/L) was only present prior to 12 of 31 interventions with paired data available.

### Patency

**Fig. 3:**
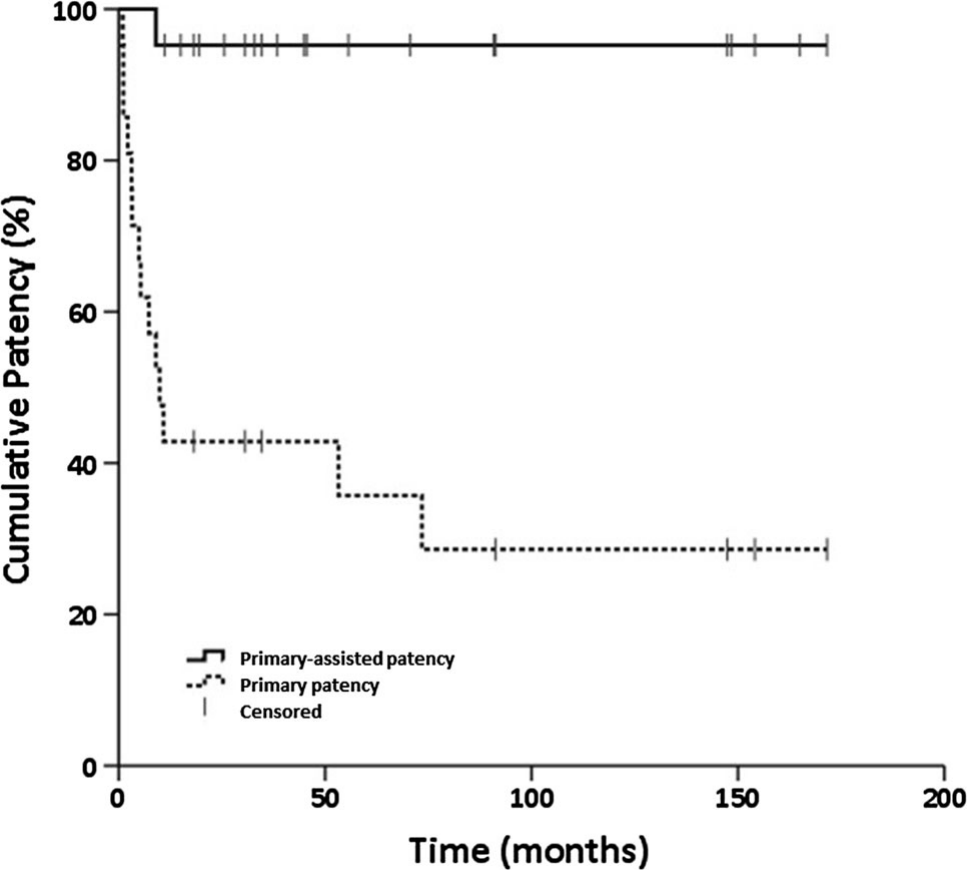
Kaplan–Meier plot of primary and primary-assisted patency

Ultrasound follow-up was available for a median of 45.5 months (range 11.1–171.6). Median primary patency was 9.9 months, with rates falling early in the study period. Primary patency was 42.9, 42.9, 35.7 and 28.6% at 1, 3, 5 and 10 years, respectively. Surveillance and re-intervention maintained good rates of primary-assisted patency. One patient had early reocclusion of a stent at 8 months, resulting in a primary-assisted patency of 95.0% at 1 year, and maintained to 10 years (Fig. 3).

### Current Patient Status

Outpatient clinical follow-up was available for a median of 65.0 months (range 13.1–189.5). All 21 patients were alive at last follow-up. Two had re-transplantation for chronic recurrent cholangitis and chronic rejection, at 12 and 25 months following the last intervention, respectively. Two had biliary strictures requiring repeated interventions. The remaining 17 are clinically well with good graft function.

## Discussion

Portal vein complications are seen more frequently in paediatric patients following liver transplantation than in adults. This is due, in part, to greater use of split liver grafts in children. In addition, biliary atresia is a common indication for paediatric liver transplantation, and the associated PV hypoplasia contributes to PVC [8–10]. In our institution, the incidence of post-transplantation PVC was 11.4% (29/255), at the higher end of reported rates [1], but comparable to those with a similar median age at transplantation [8, 14, 15].

Consistent with evidence that younger age and biliary atresia represent risk factors for development of PVC [5, 7, 9], in our study patients who developed PVC were younger than the general paediatric liver transplant population at our institution, and there was a greater proportion of patients transplanted for biliary atresia in the PVC group compared to the overall transplant population (65.5 vs. 30.8%). Younger and lower weight patients, and those with biliary atresia, have smaller recipient portal veins and are more likely to have donor-recipient vessel mismatch requiring a PV conduit, both of which are likely to predispose to PV complications [5, 9].

PVC may be found incidentally on USD surveillance or present clinically with graft dysfunction and/or complications of portal hypertension. Similar to other series, our institution’s cross-specialty consensus opinion is to actively intervene in patients with PVS, whether symptomatic or incidental, to prevent subsequent complications of portal hypertension, graft dysfunction or progression to PVT [1, 16]. We only consider intervening in asymptomatic patients if PV Doppler velocity gradient is three or more. PVT adversely affects patient and graft survival, and thrombus extending to the PV confluence makes re-transplantation more complex [9]. Furthermore, crossing a stenosis during percutaneous intervention is simpler than crossing a thrombosed PV. Our only technical failure was in a patient with PVT.

Our technique for PV intervention with either a transhepatic or transhepatic/trans-splenic approach is similar to other series [17, 18] and resulted in high-technical success (97.6%). A trans-splenic approach was only required in one of the six cases with PVT and in none of the cases with PVS. In the former case, the trans-splenic puncture was used for venography to clarify the diagnosis of PVT, which remained unclear despite a CT scan, and to assist in crossing the lesion from a combined transhepatic/trans-splenic approach. In contrast to Carnevale et al. [19], we did not routinely perform an arterioportogram, this being required in only one patient with PVT. No patients had transileocolic access.

We take a selective approach to stenting because stents will not grow with the child, and may eventually result in a ‘fixed stenosis’. Nevertheless, just over half of our patients eventually required stent placement. Future developments in stent technology, such as biodegradable stents, may change our approach. Our current preference is to use a 7-mm-diameter Palmaz Blue stent (Cordis, Baar and Switzerland), which can be dilated further to 8 mm, if required, whilst still maintaining its structural integrity. Dilatation beyond this may result in loss of hoop strength or stent fracture, but would make future insertion of a larger, adult size stent feasible [20, 21]. Being a balloon expandable stent, the Palmaz Blue can be positioned accurately, minimising the length of stent extending into the recipient PV. If too much of the stent extends into the recipient PV, this may not leave sufficient length of PV for an end-to-end anastomosis if re-transplantation becomes necessary. The patient would then require a conduit from the recipient superior mesenteric vein (SMV) onto the donor PV. Precision of stent placement also becomes important if there is subsequent stent thrombosis, since an intact splenic-mesenteric confluence leaves available the option of future surgical bypass with a meso-Rex shunt to restore hepatopetal PV flow.

The four largest comparable published series to date are summarised in Table 2 [1, 16, 17, 19], with comparator results from the present study also included. The number of interventions reported in these studies ranged from eight to 66. Technical and clinical success ranged from 76 to 100%. However, the reporting and definitions of technical success and clinical success varied between the studies, limiting direct comparison of outcomes.

**Table 2:** Comparison of the current study with other studies in the literature reporting on PTA with or without stenting for PV complications following paediatric liver transplant

Study	Number of procedures (number of patients)	Mean age at intervention (range)	Mean length of ultrasound follow-up in months (range)	Percentage of technical success (proportion)	SVS reporting standards grading of procedure-related complications [12] (CIRSE grading) [13]	Primary patency	Primary-assisted patency
Present study	42 (21)	3.1 years (4 months–16.2 years)	73.0 (11.1–171.6)	97.6% (41/42)	1 mild (grade 1) 2 moderate (both grade 3)	1 year: 43% 3 years: 43% 5 years: 36% 10 years: 29%	1 year: 95% 3 years: 95% 5 years: 95% 10 years: 95%
Yabuta et al. [1]	66 (43)	4.1 years (7 months–19 years)	107.8 (5–169)	98.4% (64/65)	2 severe (1 grade 3, 1 grade 4)	1 year: 83% 3 years: 78% 5 years: 76% 10 years: 70%	1 year: 100% 3 years: 100% 5 years: 100% 10 years: 96%
Uller et al. [17]	8 (8)	5.6 years (8 months–17.7 years)	15.2	87.5% (7/8)	None	Not reported	Not reported
Carnevale et al. [19]	15 (15)	5.1 years (1.7–15 years)	75.6 (36–97)	100% (15/15)	None	Not reported	Not reported
Funaki et al. [16]	25 (25	3.3 years (5 months–17 years)	46 (5–61)	76% (19/25)	1 mild (grade 1) 1 moderate (grade 3) 2 severe (1 grade 3, 1 grade 4)	Not reported	Not reported

Means have been calculated for data points from the current study to allow for comparison with other published studies

The largest study, with the longest follow-up, and only study reporting primary and primary-assisted patency showed primary patency of 83, 78, 76 and 70%, and primary-assisted patency of 100, 100, 100 and 96%, at 1, 3, 5 and 10 years, respectively [1]. Lower primary patency rates in our study may reflect the younger age and associated smaller PV diameter at first intervention. Difference in suturing techniques for the portal venous anastomosis may also play a role, with continuous suturing used at our centre, but interrupted suturing preferred in Japanese centres. Continuous suturing is associated with poorer compliance of vascular anastomoses [22, 23]. However, suturing technique was not reported by Yabuta et al. [1]. Our primary-assisted patency rates are comparable.

PV intervention, especially in the context of PVS, is a relatively low-risk procedure; only one of 36 of the interventions for PVS and two of six for PVT were associated with a complication. Two of these required additional medical treatment. None required additional intervention or surgery. Two of the other series do, however, describe complications directly related to the intervention requiring additional invasive procedures [1, 16].

Other published studies reporting PV intervention following paediatric liver transplantation are limited by very small numbers of cases, short durations of follow-up, combined reporting of outcomes in paediatric and adult patients or reporting of outcomes of stent placement alone [11, 24–28].

Limitations of the current study include those inherent to a retrospective case series, including incomplete data availability. Small patient numbers precludes more rigorous study designs at single institutions. Variation in technique and developments in technology over the prolonged time course of the study makes results less generalisable to future patients, though technological advances are likely to improve outcomes.

Good long-term patency and clinical outcomes are achievable with judicious surveillance following percutaneous intervention for post-transplantation PVC in paediatric patients. Future developments in biodegradable stents may overcome the issue of fixed stenoses in growing children and may improve primary patency, reducing re-intervention. Despite the need for ongoing surveillance and re-intervention, in the absence of a low-risk alternative, PTA remains the treatment of choice.
